# Skin extension with a digito-lateral flap and early active finger extension training for Dupuytren contracture: A retrospective study

**DOI:** 10.1097/MD.0000000000030130

**Published:** 2022-08-19

**Authors:** Konosuke Yamaguchi, Yoshio Kaji, Osamu Nakamura, Sachiko Tobiume, Yumi Nomura, Kunihiko Oka, Takahiro Negayama, Tetsuji Yamamoto

**Affiliations:** a Department of Orthopaedic Surgery, Faculty of Medicine, Kagawa University, Miki-Cho, Japan; b Department of Orthopaedic Surgery, Kagawa Prefectural Shirotori Hospital, Higasi-Kagawa-shi, Japan; c Department of Orthopaedic Surgery, Shikoku Medical center for Children and Adults, Zentsuji-shi, Japan.

**Keywords:** digito-lateral flap, Dupuytren contracture, early active finger extension training, orthoplastic hand surgery, surgical treatment

## Abstract

In the surgical management of Dupuytren contracture (DC), Y-V plasty (YV) and Z-plasty (ZP) are techniques often used for skin extension. However, achieving sufficient skin extension with these procedures alone is often difficult. Therefore, we addressed this issue with an adjunctive digito-lateral flap (DLF) and report the clinical results of the surgery using a DLF in addition to YV and ZP. Fifteen patients with DC (15 affected fingers) underwent partial fasciectomy using a DLF in addition to YV or ZP, and early active finger extension training was performed immediately after the operation. The flap survival rate, preoperative and postoperative extension angle, Tonkin contracture improvement (TCI) rate, and Tubiana staging grades were evaluated. The contracture sites were at 4 proximal interphalangeal (PIP) and 3 metacarpophalangeal (MP) joints of the little finger and 4 PIP and MP joints each of the ring and little fingers. All the flaps survived, and the extension angle improved at the final observation from a preoperative mean of −45° to −3° and −55° to 5° for the PIP and MP joints, respectively. One patient with PIP joint contracture treated in the early stage of the study experienced a persistent 5° limitation of extension, even though the TCI rate was satisfactory (91.9%) and the outcome was “good.” Full extension of the joints was achieved in 15 patients, in whom the TCI rate was 100% and the outcome was “very good.” This technique was able to solve 3 important steps to achieve full extension: intraoperatively, wound closure, and rehabilitation. We attained and maintained long-term full extension intraoperatively and immediately after surgery and obtained very good treatment results, as shown in this study. In conclusion, highly favorable clinical outcomes were achieved through the combination of a DLF with YV and ZP. Skin extension with a DLF is a useful surgical technique for DC.

## 1. Introduction

Surgical treatment of Dupuytren contracture (DC) has yet to achieve good results for full extension. The results are often inadequate, particularly in patients with severe flexion contractures affecting the proximal interphalangeal (PIP) joint. One of the reasons is inadequate skin extension, which prevents early active rehabilitation in the direction of extension because of the risk of wound diastasis.

In DC, skin extension is often performed using Y-V plasty (YV) or Z-plasty (ZP). However, the design of the skin incision alone is insufficient for active extension training in the early postoperative period due to inadequate skin extension and residual skin tension. As a result, we had often experienced cases in which extension restriction of the joint persists. In addition, various flaps, such as the artery perforator flap,^[1,2]^ hypothenar flap,^[3]^ and ulnar parametacarpal flap,^[4]^ have also been attempted for skin extension. Although these techniques generally yield good results, they are complicated and result in large wounds. Therefore, we believed that the use of a simpler, minimally invasive skin flap would provide a better range of motion (ROM) in extension.

A digito-lateral flap (DLF) was first used in France in 1982 by Razemon^[5]^ as a “lateral digital rotation flap” for DC. Its use for treatment of retraction after hand burns in children has also been reported in Spain.^[6]^ In Japan, a “digito-lateral flap” is described in a famous textbook by Dr. Hirase^[7]^ and is used not only to treat DC, but also for flexion contracture after trauma. DLF is a random pattern flap. The length of the flap can be extended up to the length of the crease, and there is no limit to the length-to-width ratio; thus, the flap can be created in either an anterograde or retrograde fashion. It has many additional advantages, such as its simplicity in contrast with the artery pedicle flap, which requires dissection and blood vessel elevation.

To address these problems, since 2013, we devised a technique combining a DLF with YV and ZP. By adding the DLF, which is useful for skin extension, to conventional YV and ZP, we achieved full extension immediately after surgery by adding the conventional partial fasciectomy and open contracture release where necessary.

This article introduces our technique combining a DLF with YV and ZP for DC. We also examined whether this technique would provide sufficient skin extension and good postoperative ROM for extension. In addition, the clinical results of patients who underwent this technique were evaluated.

## 2. Methods

This study was conducted with the approval of the ethics committee at our institution, and informed consent for the publication of this article was obtained from the patients. A retrospective review of patients who underwent surgical treatment for DC between April 2013 and March 2021 was conducted. The data of patients who underwent surgical treatment with partial fasciectomy, YV, and ZP plus a DLF and could be followed up for longer than 6 months were reviewed. Other inclusion criteria were ROM before surgery and at the last observation, and the timing of wound healing, as documented in the electronic medical record. Exclusion criteria were based on the patients who underwent surgery for recurrence or after injection of collagenase clostridium histolyticum.

### 2.1. Surgical technique

**Figure 1.**
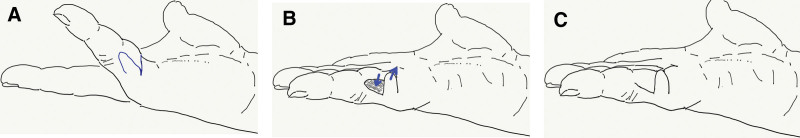
(A) The digito-lateral flap for the metacarpophalangeal joint is designed on the ulnar side of the proximal phalanx. (B) The flap is rotated from the lateral to the volar side. (C) The flap is sutured, and the metacarpophalangeal joint is fully extended.

All patients were operated under general anesthesia in the supine position with an air tourniquet. The DLFs were designed on the ulnar side of the proximal phalanx in patients with metacarpophalangeal (MP) joint disease (Fig. 1), on the ulnar side of the middle phalanx in patients with PIP joint disease, and on the ulnar side of the proximal phalanx and radial side of the middle phalanx in patients with both MP and PIP joint disease. A zig-zag skin incision was extended proximally from the MP joint; after which a YV was added, and a ZP was designed most proximally. First, an incision was made in the proximal part of the MP joint; the cord was identified and detached after the neurovascular bundle was protected, and the MP joint was extended to secure the surgical space and facilitate surgery. After that, partial fasciectomy was performed both proximally and distally while the neurovascular bundle was detached as needed. Full extension of the MP and PIP joints was checked in this state, and open contracture release was performed if this was not achieved. Hemostasis was confirmed once the tourniquet was deflated, and the wound was closed as designed. Skin deficiency did not usually occur, but the wound was closed without interfering with joint extension when it did. An artificial dermis was applied if a skin deficiency occurred on the lateral side.

### 2.2. Postoperative management

**Figure 2.**
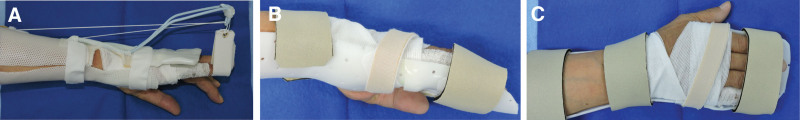
(A) Outrigger finger extension splint. (B, C) Nighttime finger extension splint.

A bulky dressing was applied on the day of surgery, while a thin dressing was applied on the following day. Active assistive exercises were started on the first day postoperatively, and the patient was actively rehabilitated using an outrigger finger extension splint and nighttime finger extension splint as orthotic therapy in the direction of the extension (Fig. 2). The tendency to bleed during the first few days postoperatively required that the dressing be changed twice a day, but changing the dressing 2 to 3 times a week was sufficient after that. The wound generally healed approximately 2 weeks postoperatively, and dressing was not needed.

### 2.3. Evaluation

Data on patient symptoms and physical findings (age, sex, site of contracture, severity, ROM at preoperative and last observation, flap survival, and duration of wound healing) were extracted from electronic medical records. We assessed Tonkin contracture improvement (TCI) rate^[8]^ of each patient as main outcomes. We also assessed the age, sex, site of contracture, severity (Meyerding classification^[9]^), the final observation extension angle at the PIP and MP joints, Tubiana criteria,^[10]^ flap survival, and duration of wound healing as secondly outcomes.

The preoperative severity was evaluated by Meyerding classification.^[9]^ ROM of the finger joint was evaluated for extension and flexion angles using a standard goniometer (Hand goniometer; SAKAI Medical Co., Ltd, Tokyo, Japan). TCI rate was calculated as the percentage of angles improved out of the preoperative angles of lack of extension. Wound healing was defined as the time when no exudate was observed and dressing was no longer required.

### 2.4. Statistical analysis

The Wilcoxon signed rank test was performed to compare the ROM between the preoperative and final values. The level of significance was set at *P* < .05. All statistical analyses were performed with EZR^[11]^ (Saitama Medical Center, Jichi Medical University, Saitama, Japan), which is a graphical user interface for R (The R Foundation for Statistical Computing, Vienna, Austria). More precisely, it is a modified version of R commander designed to add statistical functions frequently used in biostatistics. The effect size was calculated from the mean extension angles and standard deviations before and after surgery of the PIP and MP joints, and the results were 1.89 and 1.91, respectively. Therefore, the sample sizes for the PIP and MP joints were 5 and 5, respectively, when the sample size was calculated with a confidence interval of 95%. In this study, since the PIP and MP joints were 12 and 11, respectively, we considered the sample sizes to be statistically sufficient.

## 3. Results

A total of 15 cases were found to be applicable, of which all involved male patients. The mean age of the patients was 68.7 years (range, 45–78 years), and the mean follow-up duration was 14.9 months (6–30 months).

The contracture sites were at 4 PIP and 3 MP joints of the little finger and 4 PIP and MP joints each of the ring and little fingers.

The preoperative severity was grade 1 in 5 fingers, grade 2 in 1 finger, and grade 3 in 9 fingers.

All the flaps survived, and wound healing averaged 21.1 days (14–50 days); delayed healing occurred in 1 patient with diabetes mellitus, but that patient’s wound finally healed in 50 days.

Preoperative and final extension angles significantly improved from an average of −49.8° (−10° to −90°) to −0.4° (−5° to 10°) in the PIP joint and from an average of −51.8° (−20° to −90°) to 6.4° (0° to 10°) in the MP joint (Table 1). One patient in the early stage of this series whose PIP joints were affected had a −5° limitation of extension and a TCI rate of 93.4%, and the outcome was “good”, whereas the remaining 3 patients had a TCI rate of 100% and the surgical outcome was “very good”. In the remaining 2 patients with MP joint disease and 8 with both PIP and MP joint disease, full extension of the joints was attained in all cases, the TCI rates were 100%, and the postoperative outcomes were “very good.”

**Table 1:** Clinical results.

Operation site (n)		Finger (n)	Flap survival	Pre-op (°)		Final obs (°)		TCI rate (%)
				PIP ext	MP ext	PIP ext	MP ext	
PIP	(4 cases)	Small (4)	All	−76.8		−1.3		98.4
MP	(3 cases)	Small (4)	All		−66.7		3.3	100.0
PIP + MP	(8 cases)	Ring (4)Small (4)	All	−36.3	−46.3	0.0	7.5	100.0
Average				−49.8	−51.8	−0.4	6.4	99.6

ext = extension, MP = metacarpophalangeal, PIP = proximal interphalangeal, obs = observation, Pre-op = preoperative, TCI = Tonkin contracture improvement.

**Figure 3.**
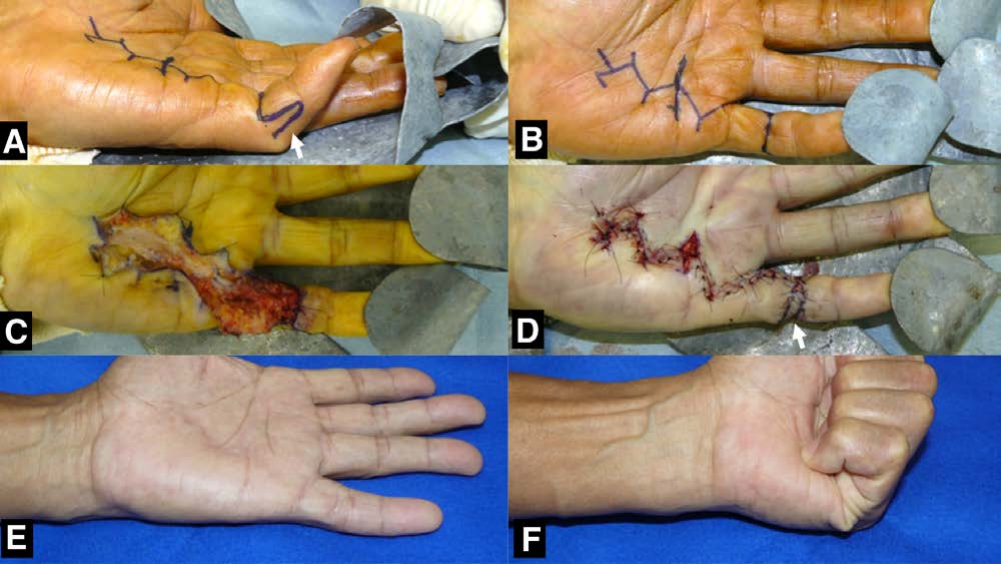
Dupuytren contracture of the left little finger and contracture of the PIP joint. (A, B) The PIP joint is severely limited in extension (−62° in extension), and a digito-lateral flap (arrow) for the PIP joint is designed perioperatively on the ulnar side of the middle phalanx, and the Y-V and Z-plasties are designed on the palmar side. (C, D) A partial fasciectomy is performed, and the Y-V and Z-plasties and digito-lateral flap (arrow) are closed as designed, and the skin on the palmar side is extended. (E, F) The outcome is good at 6 mo postoperatively: extension, −5° and flexion, 95°. PIP = proximal interphalangeal.

A representative case with images during the surgery and 6 months after the surgery are shown in Figure 3.

## 4. Discussion

Partial fasciectomy is the standard treatment for DC.^[12–14]^ However, there are some cases with poor results. This is especially true in patients with little finger and PIP joint disease.^[15]^ The main reason for poor results is the lack of initial correction in the ROM of extension.^[16]^ Skin extension and postoperative therapy have been devised for this reason.

In the surgical treatment of DC, we consider 3 important steps that need to be resolved in order to achieve full extension. These 3 steps are as follows: intraoperative, wound closure, and rehabilitation.

First, to achieve full extension intraoperatively, no cord that inhibits extension should remain and the joint contracture should be released. To ensure reliable resection of the cord, we use a microscope for dissection between the spiral cord and neurovascular bundle, if necessary. If full extension is not achieved after the cord is resected, we perform an open contracture release procedure. Specifically, we perform the following procedures in this order: removal of the swallow tail, volar plate, and fan-like portion of the collateral ligament, after which full extension is achieved.

In the second step, the lack of skin must be resolved. This is because even if full extension of the joint is achieved in the first step, full extension will not be achieved if the wound is closed with high tension in the palmar skin. In other words, only closing the skin is not sufficient. Various techniques have been attempted previously, such as Burner volar zig-zag,^[17,18]^ open palm technique,^[19,20]^ the “Jacobsen flap” technique,^[21]^ multiple ZP,^[22]^ multiple V-Y plasty,^[18]^ YV and ZP,^[23]^ skin grafting,^[24,25]^ artery perforator flap,^[1,2]^ hypothenar flap,^[3]^ ulnar parametacarpal flap,^[4]^ and DLF,^[5]^ among others. In our department, we initially performed YV and ZP, but we later used a DLF, which is the simplest and least invasive of these techniques for closing the wound, in combination with YV and ZP.

This section discusses the rate of improvement in contracture depending on the method of wound closure. Adam and Loynes^[15]^ reported that the rate of contracture improvement in the PIP joint of the little finger is prone to poor results. Uemura et al^[23]^ reported that the rate of contracture improvement is approximately 40% in YV and ZP. However, the rate of contracture improvement in the present study with DLF was 99.2%, which is excellent compared to the results of previous reports. Moreover, compared with the rate of contracture improvement in other flaps, the rate of hypothenar flap, which was reported by Kojima et al,^[3]^ or the rate of ulnar parametacarpal flap, which was reported by Hirase et al^[4]^ ranged between 98% and 100%. Further, the results of the present study were equivalent or better for the MP joint compared to these studies. One advantage of DLF over these flaps is that the DLF can be used both for the MP and PIP joints and in combination. On the other hand, the hypothenar flap and ulnar parametacarpal flap are both far from the PIP joint. One of the disadvantages that is a concern is necrosis of the flap. However, there is a risk compared to regular VY and ZP, but in this study, no necrosis was observed even in a single case. Furthermore, compared to other flaps, the risk of necrosis is low because it is simple and does not require dissection of the blood vessels, and the amount of flap movement is less.

As a third step, postoperative therapy is important to maintain the fully acquired extension of the joint. The previous standard procedure was to immobilize the patient in the extended position for several days postoperatively and then start rehabilitation. However, in the occupational therapy program at our department, the wound is dressed the day after surgery, active assistive exercises are started early, and outrigger finger extension braces and nighttime finger extension braces are used as orthotic therapy. Since the wound is closed with no skin tension in the second stage, active rehabilitation in the direction of extension can be performed without fear of the wound tearing. With these innovations, we can attain full extension immediately after surgery, maintain it for long term, and obtain very good treatment results, as shown in this study.

Treatment of DC using collagenase injection has been reported recently.^[26–28]^ The rate of contracture improvement with collagenase injection is good in the MP joints but remains poor in the PIP joints.^[28]^ In particular, in a report by Van Beck et al that described cases with a preinjection insufficient extension angle of 20° or more, over 60% of all cases had contractures of 20° or more in extension^[29]^ Based on these reports, our department policy was “to actively consider surgery for patients with insufficient extension (contracture) of the PIP joint exceeding 30° if the patient wishes to achieve full extension.” However, since collagenase injections are not available in our country, surgery using a DLF remains the first choice to treat DC in both the MP and PIP joints in our department.

The limitations of this study are the small number of cases and short observation period. Recurrence was not observed at 6 months postoperatively, but it remains possible under long-term observation. Therefore, further observation is necessary. Another limitation is the wide age range of the selected patients, which may be influenced by aging.

In conclusion, the DLF was used in combination with the open contracture release procedure as needed to achieve full extension intraoperatively and maintain it. In addition, early active ROM training could be performed from the day after surgery without fear of wound dehiscence. In comparison with other types of skin flaps, this is a simple method and the results were very good. As collagenase injection is not available in our country, this surgical method using a DLF is a useful method for treating DC.

## Author contributions

**Conceptualization:** Konosuke Yamaguchi, Yoshio Kaji, Data curation: Konosuke Yamaguchi.

**Formal analysis:** Konosuke Yamaguchi.

**Investigation:** Konosuke Yamaguchi, Kunihiko Oka, Osamu Nakamura, Sachiko Tobiume, Takahiro Negayama, Yoshio Kaji, Yumi Nomura.

**Methodology:** Konosuke Yamaguchi, Osamu Nakamura, Yoshio Kaji.

**Project administration:** Konosuke Yamaguchi, Tetsuji Yamamoto, Yoshio Kaji.

**Resources:** Konosuke Yamaguchi.

**Software:** Konosuke Yamaguchi.

**Supervision:** Konosuke Yamaguchi, Osamu Nakamura, Yoshio Kaji.

**Validation:** Konosuke Yamaguchi, Kunihiko Oka, Yoshio Kaji.

**Visualization:** Konosuke Yamaguchi.

**Writing – original draft:** Konosuke Yamaguchi.

**Writing – review & editing:** Konosuke Yamaguchi.
